# Impacts of the Covid-19 pandemic on smoking cessation success

**DOI:** 10.4314/ahs.v23i3.50

**Published:** 2023-09

**Authors:** Pakize Ayse Turan, Onur Turan

**Affiliations:** 1 Menemen State Hospital, Chest Diseases Department, Izmir — Turkey; 2 Izmir Katip Celebi University Atatürk Research and Training Hospital, Chest Diseases Department, Izmir-Turkey

**Keywords:** Smoking, cessation, pandemics

## Abstract

**Setting-objective:**

Current COVID-19 outbreak has led to many behavioural changes, including smoking behaviours. We aimed to investigate the success of quitting smoking of smoking cessation outpatients.

**Design:**

Patients who applied to the smoking cessation outpatient clinic of a state hospital during the pandemic were retrospectively analysed. Smoking cessation success, personal views and experiences about COVID-19 were questioned. Hospital Anxiety and Depression (HAD) Scale was applied.

**Results:**

The smoking cessation rate in the study population was 14.7%. The reasons for not being able to quit smoking were; stress (51.9%), drug discontinuation (28.4%) and reasons related to COVID-19 (12.3%). According to HADS scores; 35.8% of the participants were at risk for anxiety and 72.6% for depression. Those with pulmonary symptoms at the time of application (p=0.001), those who continued smoking cessation treatment (p=0.016), and those without depressive symptoms (p=0.040) were significantly more successful in quitting smoking. The rate of continuing smoking was significantly higher in patients with a history of COVID-19 <18.9% of participants>(p=0.013).

**Conclusion:**

Intense stress and depressive symptoms, discontinuation of smoking cessation treatment and being infected with Coronavirus negatively affect the smoking cessation process in pandemic. These parameters should be considered during smoking cessation interviews and behavioural support should be obtained if necessary.

## Introduction

Coronavirus disease (COVID-19), which was first detected in China in December 2019, has rapidly spread worldwide. Since December 2020, severe acute respiratory syndrome coronavirus 2 (SARS-CoV-2) has infected over 642 million people, with more than 6,600,000 deaths globally^1^.

Despite the wide impact of COVID-19 on people's lives, there is a limited information about the impact of pandemic on addiction-related behaviours, especially on smoking 2. It is not clear whether the addiction to tobacco products have increased or decreased in pandemic. There have been different factors associated with the COVID-19 pandemic which may have variable impacts on tobacco addiction.

COVID-19-related lockdowns and movement restrictions, with global economical and financial constraints may reduce access to tobacco products, which may help to reduce smoking 3. Since COVID-19 is a disease that commonly affects the lungs, people may have reduced smoking because they thought smoking would increase damage to the lungs.

On the other hand, experiencing COVID-19 pandemic may have a negative psychological impact, which may induct negative emotions and stress 4. According to most smokers, smoking provides relief. So, these emotional states may lead to increased tobacco consumption 5.

There are only a few studies assessing the impact of COVID-19 outbreak on smoking behaviors, which have conflicting results. While a 3% reduction in smoking was reported due to the pandemic 6, there is also a publication reporting that a quarter of the participants reduced their smoking and motivated more than a third to quit smoking^7^.

Thus, the overall impact of the COVID-19 pandemic on smoking habits in the general population is unclear. Therefore, it is very difficult to predict the success of people who apply to smoking cessation outpatient clinics in quitting smoking. We aimed to investigate the success of quitting smoking and the factors affecting this situation in patients followed by the smoking cessation outpatient clinic during the pandemic.

## Methods

We conducted a single-center, cross-sectional study on patients admitted to the Menemen State Hospital Smoking Cessation Clinic between June 2020 and March 2021. The records of 187 patients, who have passed at least 6 months since the smoking cessation interview, and registered on Tobacco Addiction Treatment Monitoring System (TUBATIS) were retrospectively reviewed. Each patient had been called by phone for follow-up and questionnaire. Of the 187 patients contacted by phone, a total of 95 patients who agreed to participate, responded to the online questionnaire, gave verbal consent and who were over 18 years of age were included. Patients who refused to participate in the study and did not answer our phone calls we made at three different times were excluded.

Demographic information, smoking cessation habits and parameters related to their treatment were recorded from the hospital system.

Questionnaire included 23 questions, administered over the phone. First 6 questions were about demographic features and smoking habit. Participants who declared not to smoke for at least 6 months were considered to have quit smoking.

Their personal views and experiences about COVID-19 were questioned between question 7 and 9. Real-time PCR (RT-PCR) results of participants were recorded from public health management system to confirm if they had the diagnosis of COVID-19 or not.

There were questions of Hospital Anxiety and Depression (HAD) Scale beginning with question 10. HADS, developed by Zigmond and Snaith, is a self-report scale to determine the risk of anxiety and depressive states among medical patients 8. The validity and reliability of the Turkish version of the HADS was developed and tested by Aydemir et al 9. It includes 14 questions; anxiety is evaluated by odd and depression by even-numbered questions. Each question has a 4-point (0–3) response category, and possible scores range from 0 to 21 for anxiety and 0 to 21 for depression. Two cut-off scores are used for detecting depression and anxiety; a score of 11 or higher is a valid case for anxiety, and it is 8 or higher for depression. All participants consented before responding to the questionnaire, were able to terminate their participation at any point, provided their responses anonymously.

Ethics committee approval for the study was obtained from the Ethics Committee of the Izmir Katip Çelebi University Atatürk Training and Research Hospital, (Approval date and number: 21.10.2021/ IRB # 0430) and permission for the study was obtained also from the Ministry of Health of the Republic of Turkey.

## Results

Of 95 patients, 51 male (53.7%) and 44 female (46.3%), the mean age was 40.3 ± 10.1. There was at least one comorbidity in 38 patients (40%). Participants smoked an average of 28±10 packs of cigarettes per year.

The most commonly used smoking cessation treatment was varenicline (94.7%), and the other options were: 2.1% bupropion, 2.1% nicotine patch, 1.1% nicotine gum. 35.8% of the patients completed their treatment to 3 months by taking continuation packs. There were 67 participants (70.5%) who used their smoking cessation drugs regularly.

The smoking cessation rate in the study population was 14.7%. While 65.3% of patients used the same number of cigarettes, 20% had a decrease in the number of cigarettes.

**Figure 1:**
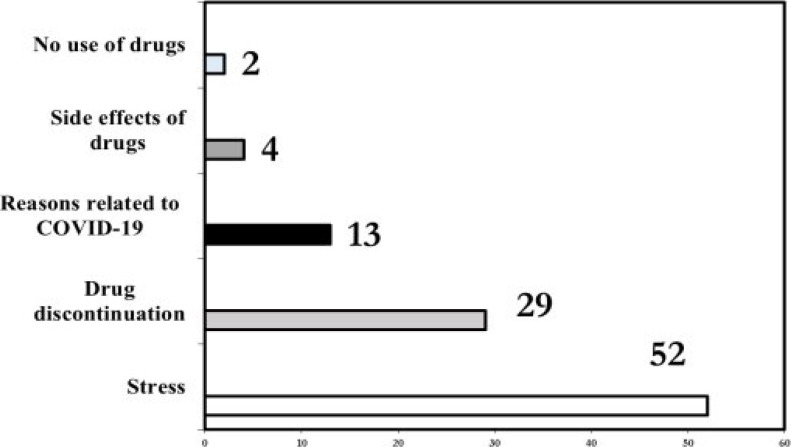
Reasons for failure to quit smoking *Drug discontinuation: This term refers to not receiving the continuation treatment from the cessation center or pharmacy of the started smoking cessation medicine

51.6% of the participants stated that the presence of the pandemic had a negative effect on the smoking cessation process. The most common reasons for not being able to quit smoking were; stress (51.9%), drug discontinuation (28.4%) and reasons related to COVID-19 (12.3%) (figure 1).

18.9% of the participants were diagnosed with COVID-19. and 32.6% had a relative infected by SARS-CoV-2.

According to HADS scores; 35.8% of the participants were at risk for anxiety and 72.6% for depression. The mean score of HADS for anxiety was 8.6 ± 4.6, and foi depression was 9 ± 3.4.

Those with pulmonary symptoms at the time of application (p=0.001), those who continued smoking cessation treatment (p=0.016), and those without depressive symptoms (p=0.040) were significantly more successful in quitting smoking (Table 1).

**Table 1:** Clinical features of participants according to success at smoking cessation

Features	Successful in smoking cessation (n=14)	Unsuccessful in smoking cessation (n=81)	p value
Gender (male/female)	6/8	45/36	0.379
Age (mean)	44.00±10.93	39.67±9.82	0.137
Comorbidities (present/not)	5/9	33/48	0.723
Educational status (below high school / not)	12/2	54/27	0.131
Cigarette (package/year)	26.00±8.24	28.28±10.48	0.697
Pulmonary symptoms at the time of application (present/not)	10/4	20/61	0.001*
Continued smoking cessation treatment (yes/no)	9/5	25/56	0.016*
Fagerstrom score (mean)	8.07±1.21	8.79±1.46	0.084
Depressive symptoms (present/not)	7/7	62/19	0.040*
HADS-A score (mean)	9.36±3.08	8.46±2.11	0.650
Anxiety symptoms (present/not)	4/10	30/51	0.387
HADS-D score (mean)	9.57±3.75	8.95±2.13	0.644
History of COVID-19 (present/not)	6/8	12/69	0.013*

* Statistically significant

The rate of continuing smoking was significantly higher in patients with a history of COVID-19. (88.9% vs. 84.4%, p=0.013). (Table 2).

**Table 2:** Relationship between smoking cessation and presence of COVID-19

History of COVID-19	Successful in smoking cessation (n=14)	Unsuccessful in smoking cessation (n=81)	p value
COVID-19 present **n, %**	2, 11.1%	16, 88.9%	0.013*
COVID-19 not present **n, %**	12, 15.6%	65, 84.4%	

* Statistically significant

## Discussion

We conducted a single-center, cross-sectional study on 187 patients who admitted smoking cessation clinic during pandemic. A questionnaire including their status and views about smoking cessation, psychological condition and COVID-19 was applied to 95 participants, who were contacted by phone. Smoking cessation rate in the study population was 14.7%. Intense stress and depressive symptoms, discontinuation of smoking cessation treatment and being infected with Coronavirus negatively affect the smoking cessation process in the pandemic. We determined that 14.7% of the patients quit smoking in our study. There are a few studies about smoking cessation during pandemic in the literature. Tetik et al. revealed the smoking cessation rate as 23.7% before pandemic, while it was found as 31.1% during pandemic 10. Another study reported that the rate of quitting smoking was 13.3% in COVID-19 outbreak 11. Since the tendency to quit smoking is constantly changing during the pandemic process, different studies have found different rates of smoking cessation success.

Stress from the COVID-19 and economic crises were found as the highest reported reason for smoking behavior change during pandemic 12. Stress about COVID-19 may have a mixed effect on smoking behavior changes. High stress levels are associated with an increased prevalence of smoking and low smoking cessation rates in many studies 13,14. On the other hand, the pandemic and the increased risk of being infected by Sars-CoV2 may cause a positive behavior modification such as decreasing smoking 15, 16. In our study, stress was reported as the main reason for their unsuccessful smoking cessation attempts by participants, similar to other many studies.

Participants with pulmonary symptoms at the time of application to smoking cessation clinic were found to be more successful in quitting smoking. Pulmonary symptoms like frequent cough, phlegm and shortness of breath were associated with intention to quit smoking in the next one month according to a study 17. Someone with complaints such as shortness of breath and cough may be more motivated to quit smoking, thinking that they will have lung diseases such as COPD.

The presence of depressive symptoms has been known to negatively affect tobacco quitting attempts. Glassman et al. reported that smokers with unsuccessful attempts at quitting smoking had significantly higher depression scores than those who were successful at smoking cessation 18. Coronavirus disease is a public health problem that causes negatively affect the psychological state, which may generally increase depressive symptoms 19. Therefore, we think that negative mood during the pandemic period will negatively affect smoking cessation.

On the other hand, smokers tend to be more anxious and tense, who show more traits of neuroticism and psychoticism than do ex-smokers or non-smokers 20. Psychiatric disorders such as anxiety and depression were observed more frequently in people with high-grade nicotine addiction 21. The presence of the pandemic may also have increased the depressive symptoms in our patient group consisting of smokers.

The rate of continuing smoking was significantly higher in patients with a history of COVID-19. Our low treatment completion rate (35.8%) may also be due to pandemic. Tobacco smoking is a known risk factor for many respiratory infections, including COVID-19. It has been reported that the current COVID-19 disease is more fatal in smokers 10. Besides, smokers are more likely to develop severe disease with COVID-19, compared to non-smokers 22. However, it is thought that those who have had COVID-19 could not continue their decision to quit smoking due to the stress and negative emotions they experienced during the illness.

This research has some limitations. First, a limited number of patients were included in the study. Another limitation is due to the self-report nature of the questionnaire. Further research using direct observational approaches with a high number of participants may yield additional evidence of the impact of the COVID-19 pandemic on tobacco addiction.

## Conclusion

The current COVID-19 outbreak has led to many behavioral changes, including smoking behaviors. Intense stress and depressive symptoms, discontinuation of smoking cessation treatment and being infected with Coronavirus negatively affect the smoking cessation process in the pandemic. These parameters should be considered during smoking cessation interviews and behavioral support should be obtained if necessary.
